# Are children living with obesity more likely to consult their general practitioner for knee pain? Longitudinal analysis of linked primary care and National Child Measurement Programme (NCMP) records.

**DOI:** 10.23889/ijpds.v7i3.1824

**Published:** 2022-08-25

**Authors:** Nicola Firman, Kate Homer, Gill Harper, John Robson, Carol Dezateux

**Affiliations:** 1 Queen Mary University of London

## Objectives

More than one in four 11-year-old children in England are living with obesity. The implications for future musculoskeletal health remain unclear. We assessed whether general practitioner consultations for knee pain were more likely among children with obesity, and how this varied by sex, ethnic background, and area-level deprivation.

## Approach

Of 61,478 11-year-old NCMP participants (2013-19), we linked 60,723 (98.8%) to their primary care records. 58,761 children (50.9% male) had no recorded knee pain consultation (including arthralgia and Osgood-Schlatter’s disease) prior to the NCMP measurement date. We calculated the proportion with a consultation for knee pain by ethnic-adjusted weight status (underweight<2nd; overweight≥91st; obese≥98th centile), sex, ethnic background and Index of Multiple Deprivation quintile. We studied time to first general practitioner consultation for knee pain after the NCMP date by fitting Cox proportional hazards models, estimating mutually-adjusted hazard ratios (aHRs) and 95% confidence intervals (CI) for boys and girls separately.

## Results

We identified 2503 (4.3%) children with at least one consultation for knee pain after the NCMP date. Boys were more likely to consult than girls (mean difference 1.5%; 95% CI: 1.2,1.9). Median time to first knee pain consultation was 1.88 years (IQR: 0.94,2.93). In adjusted analyses, boys with underweight (aHR 0.16; 95% CI: 0.05,0.51), from South Asian ethnic backgrounds (0.80; 0.69,0.92) and living in less deprived areas (Wald test statistic: 11.41; p-value=0.0223) were less likely, and those from Black ethnic backgrounds (1.31; 1.13,1.51) more likely, to consult with knee pain. Girls from South Asian ethnic backgrounds (0.70; 0.59,0.84) and those living in less deprived areas (15.44; p-value=0.0039) were less likely, and those with a BMI considered obese (1.30; 1.10,1.54) more likely to do so.

## Conclusion

Adolescent girls, but not boys, living with obesity are more likely to consult their general practitioner with knee pain. Ethnic differences in knee pain consultations merit further study. Linkage of primary care and NCMP records enables greater understanding of health service utilisation by children by weight status and demographic characteristics.

